# Comparative assessment of oral health knowledge and dental behavior among individuals with and without hearing impairments in Jordan

**DOI:** 10.3389/froh.2025.1643903

**Published:** 2025-09-19

**Authors:** Sabha Mahmoud Alshatrat, Wael Mousa Al-Omari, Abedelmalek Kalefh Tabnjh, Majd M. Alsaleh, Siddharthan Selvaraj

**Affiliations:** 1 Department of Applied Dental Sciences, College of Applied Medical Sciences, Jordan University of Science and Technology, Irbid, Jordan; 2 Department of Prosthodontics, Faculty of Dentistry, Jordan University of Science and Technology, Irbid, Jordan; 3 Department of Cardiology, Odontology School, Sahlgrenska Academy, Gothenburg University, Gothenburg, Sweden; 4 Dental Research Unit, Center for Global Health Research, Saveetha Medical College and Hospital, Saveetha Institute of Medical and Technical Sciences, Chennai, India; 5 Department of Pediatric Dentistry, University of Illinois Chicago, Chicago, IL, United States; 6 Faculty of Dentistry, University of Puthisastra, Phnom Penh, Cambodia; 7 Department of Dental Research Cell, Dr. D. Y. Patil Dental College & Hospital, Dr. D. Y. Patil Vidyapeeth (Deemed to be University), Pune, India

**Keywords:** oral care, hearing impairments, dental behavior, oral health knowledge, deaf

## Abstract

**Background:**

Individuals with hearing impairments face significant barriers in accessing oral health information, which may negatively influence their oral hygiene practices and outcomes. This study aimed to compare oral health knowledge and dental behavior between individuals with and without hearing impairments in Jordan.

**Methods:**

A cross-sectional comparative study was conducted among 289 participants, comprising 149 individuals with hearing impairments and 140 individuals without hearing impairments. A validated, self-designed closed-ended questionnaire assessed participants’ oral health knowledge and behaviors. Convenience sampling was employed to recruit participants from centers associated with hearing impairment. Data were analyzed using SPSS® version 22, with a significance level set at *P* < 0.05. Chi-square tests and contingency table analyses assessed group differences.

**Results:**

Individuals with hearing impairments demonstrated significantly less knowledge regarding toothbrush hardness, frequency of brushing, the importance of routine dental visits, and recognition of gum disease signs (*P* < 0.05). A smaller proportion of the hearing-impaired group brushed their teeth once or twice daily (82.8% vs. 93.3%, *P* < 0.001), used dental floss and mouthwash, consumed soda more frequently (*P* < 0.001), and reported using fluoridated toothpaste compared to individuals without hearing impairments (*P* < 0.05).

**Conclusions:**

The substantial gaps in oral health knowledge and practices among individuals with hearing impairments highlight the need for targeted educational interventions. Culturally sensitive tools, such as visual aids, videos, and tailored oral health programs, could improve hygiene practices, reduce disease risk, and enhance the quality of life in this underserved population.

## Introduction

Hearing impairment is the third most common disability worldwide (1). Its etiology may be hereditary, congenital, or acquired, and it can present as conductive, sensorineural, mixed, or central hearing loss. The severity of hearing impairment ranges from mild (less than 40 decibels) to profound (greater than 40 decibels), and it may occur prelingually, perilingually, or postlingually, depending on the timing relative to language acquisition (2). According to the World Health Organization (WHO), over 5% of the global population—432 million adults and 34 million children—live with hearing loss that requires intervention. By 2050, this number is projected to rise to over 700 million, affecting approximately 1 in 10 people (3).

Studies have consistently demonstrated that individuals with hearing impairments exhibit poorer oral hygiene compared to the general population, primarily due to limited access to oral health information and communication barriers (4, 5). Bhadauria et al.'s study (6), conducted in India, surveyed 500 individuals with hearing difficulties and found that they had a 30% higher prevalence of caries compared to the general population. Cannobbio et al.'s study, conducted in Italy, found that individuals with hearing difficulties were 20% less likely to utilize preventive dental services compared to those without hearing impairments.

Because health information is frequently delivered verbally, people with hearing loss may not receive or fully comprehend oral health guidance, contributing to lower health awareness and higher rates of oral disease (7, 8). In particular, difficulties in understanding dental terminology, oral hygiene practices, and the importance of routine care hinder their ability to maintain their oral health. Every individual has unique sensing skills, which influence their degree of understanding. Studies from high-income countries demonstrate that children with hearing impairments have significantly poorer oral hygiene than their hearing peers. A systematic review reported mean plaque and gingival index scores of 0.99 and 1.27, respectively, among individuals with hearing loss (3). Visually based interventions have shown efficacy in improving outcomes—for example, a UK-based randomized controlled trial (RCT) demonstrated that video instruction reduced plaque and gingival indices by 0.37 and 0.39, respectively, in this population (9).

Oral health knowledge, which encompasses understanding the significance of regular brushing, appropriate toothbrush selection, brushing techniques, dietary habits, and the importance of routine dental visits, is crucial for maintaining good oral health. Communication barriers often hinder individuals with hearing impairments from obtaining this information effectively (10). Health promotion strategies, including the use of visual aids and tailored educational programs, have been shown to improve knowledge and facilitate positive behavior change (11, 12). A randomized controlled trial in the UK demonstrated that visual instruction significantly reduced plaque and gingival indices in children with hearing impairments (13).

Despite the recognized link between oral health literacy, behavior, and clinical outcomes, there is limited data on the oral health knowledge and practices of individuals with hearing impairments in Jordan. Dentists may also encounter challenges in providing care to this group due to communication difficulties and limited training in accommodating their needs.

This study aims to assess oral health knowledge and dental behavior among individuals with hearing impairments compared to those without hearing impairments in Jordan. The findings are expected to provide a baseline for developing targeted interventions that enhance oral health awareness and outcomes in this underserved population.

## Materials and methods

A researcher-developed questionnaire in Arabic, the official language in Jordan, was used to assess oral health knowledge and dental behavior among individuals with hearing impairments. The content validity of the questionnaire was established by two professional experts. The average congruency percentage (ACP) was calculated, which reflects the level of agreement among the expert panel members. The ACP was 92%, which indicates that the questionnaire was deemed to be valid by the professional experts and the items of the questionnaire were relevant and appropriate for this study. The consistency of the questionnaire was assessed by test–retest reliability, which was done by administering the same questionnaire twice to the same participants (*n* = 10). The internal reliability of the questionnaire was assessed using Cronbach's alpha coefficient, with an acceptable internal consistency of 0.75, indicating that the items in the questionnaire are related to each other. A pilot test for this study was conducted by administering the questionnaire to 10 caregivers of individuals with hearing impairments to provide feedback on the questionnaire’s content, clarity, and format. The questionnaire includes three parts. The first part focused on demographic information and included five items related to basic demographic information. In the second part, there were 12 items that evaluated the participant's oral health knowledge. Finally, in the third part, there were seven items that assessed the participant's dental behavior. The severity of a participant’s hearing impairment was objectively classified based on diagnostic records obtained from files provided by the school’s or center’s administrators. Thus, the severity was not self-reported but determined by professional clinical examination and documented in official records. Participants with hearing impairments were recruited from special-care centers and schools across Jordan through convenience sampling, which may have introduced selection bias. The participants were required to have basic reading ability in Arabic as part of the inclusion criteria to ensure consistent understanding and independent completion of the questionnaire.

The institutions were identified through the Ministries of Social Development and Education. Participation was contingent on the willingness of the administrators to distribute the questionnaire. A printed copy of the questionnaire and a cover letter explaining the study were distributed to the participants. Individuals who were literate completed the form themselves, while caregivers assisted those who were not. A group of individuals without hearing impairments was recruited from the same geographical areas using convenience sampling, which may have introduced selection bias. They were not matched by age or socioeconomic status, which may have affected comparability between the groups.

G*Power Sample Size Calculator software was used for the sample size calculation, with an 80% power and a margin of error of 5% based on Alshatrat et al. (14). The minimum sample size required for this study was 139 for each group.

A total of 200 questionnaires were sent to individuals with hearing impairment, of which 149 were completed and returned. In addition, 140 completed questionnaires from the individuals without hearing impairment were returned out of the 200 sent.

Data were collected and analyzed using the IBM SPSS Statistics for Windows Version 25.0 (IBM Corp., NY, USA; https://www.ibm.com). We conducted a descriptive statistical analysis of the sociodemographic characteristics and responses to the questions. The results are presented in terms of numbers and proportions. Intergroup comparisons were performed using two-tailed chi-square tests. Results were considered statistically significant at *p* < 0.05.

## Results

The study included 289 participants, with 149 individuals with hearing impairments (HI group) and 140 individuals without hearing impairments. The mean age of the participants was 15 years, with most participants under 18 years. In the HI group, 57.1% had a severe hearing impairment, and 32.9% had a moderate impairment. Additional sociodemographic characteristics, including gender distribution, education level, and family income, are detailed in Table 1.

**Table 1:** Sociodemographic characteristics of the participants with hearing impairments and those without hearing impairments.

Characteristic	Hearing-impaired group, *n* (%)	Without hearing impairment group, *n* (%)
Sex		
Male	73 (52.1)	100 (67.1)
Female	67 (47.9)	49 (32.9)
Age		
<18	123 (87.9)	149 (100)
18–40	13 (9.3)	0 (0)
>40	4 (2.9)	0 (0)
Education		
Elementary	96 (68.6)	132 (88.6)
Middle school	32 (22.9)	17 (11.4)
High school	7 (5)	0 (0)
College and higher	4 (2.8)	0 (0)
Family income (JD)		
<250	71 (50.7)	2 (1.3)
250–500	63 (45)	70 (47.0)
500–1,000	4 (2.9)	64 (43.0)
>1,000	2 (1.4)	13 (8.7)
Insurance		
Yes	95 (67.9)	100 (67.1)
No	45 (32.1)	49 (32.9)

Regarding oral health knowledge, the analysis revealed no statistically significant differences between the HI group and those without hearing impairments in their understanding of plaque formation, caries etiology, the effects of sugar and soft drinks, and the relationship between oral health and general health (*P* > 0.05). However, notable differences were observed in other areas.

The individuals with hearing impairments were significantly less likely to know that gums should not bleed or swell during brushing (*P* < 0.001). They also demonstrated poorer knowledge regarding toothbrush hardness, with 77.1% of the HI group incorrectly believing that a hard toothbrush is needed to clean teeth, compared to only 4.7% of the individuals without hearing impairments (*P* < 0.001). In addition, 54.3% of the HI group thought dental visits were only necessary in the presence of a toothache, compared to 21.5% of the individuals without hearing impairments (*P* < 0.001). Notably, the HI group showed higher awareness regarding the necessity of dental floss use (87.9% vs. 75.8%, *P* = 0.019), possibly reflecting targeted health messages at the centers from which the participants were recruited. Full details of the oral health knowledge responses are presented in Table 2.

**Table 2:** Oral health knowledge among individuals with hearing impairments compared to those without hearing impairments.

Questions	Hearing impaired, *n* (%)	Without hearing impairments, *n* (%)	*P*-value
1. Dental plaque is formed by colonizing bacteria			
Yes	66 (48.5)	90 (60.4)	0.069
No	9 (6.6)	4 (2.7)	
Do n't know	61 (44.9)	55 (36.9)	
2. Dental caries are mainly caused by bacteria			
Yes	95 (67.9)	117 (79.1)	0.073
No	22 (15.7)	18 (12.2)	
Do n't know	23 (16.4)	13 (8.8)	
3. Eating sugar can lead to dental caries			
Yes	135 (98.5)	140 (94.0)	0.080
No	1 (0.7)	8 (5.4)	
Do n't know	1 (0.7)	1 (0.7)	
4. Drinking soft drinks affects dental health			
Yes	124 (88.6)	141 (94.6)	0.126
No	7 (5.0)	5 (3.4)	
Do n't know	9 (6.4)	3 (2.0)	
5. Is there any relationship between oral health and overall health?			
Yes	111 (79.9)	125 (83.9)	0.620
No	19 (13.7)	15 (10.1)	
Do n't know	9 (6.5)	9 (6.0)	
6. It is normal for your gums to bleed during brushing			
Yes	58 (42.0)	31 (20.8)	*0*.000*
No	62 (44.9)	112 (75.2)	
Do n't know	18 (13.0)	6 (4.0)	
7. It is normal for your gums to be red			
Yes	16 (11.4)	49 (32.9)	*0*.000*
No	119 (85)	89 (59.7)	
Do n't know	5 (3.6)	11 (7.4).	
8. It is normal for your gums to be swollen			
Yes	120 (85.7)	5 (3.4)	*0*.000*
No	19 (13.6)	139 (93.3)	
Do n't know	1 (0.7)	5 (3.4)	
9. Brushing your teeth regularly protects your teeth			
Yes	120 (86.3)	142 (95.3)	*0*.021*
No	16 (11.5)	6 (4.0)	
Do n't know	3 (2.2)	1 (0.7)	
10. You need to visit a dentist only when you have a toothache			
Yes	75 (54.3)	32 (21.5)	*0*.000*
No	31 (22.5)	116 (77.9)	
Do n't know	32 (23.2)	1 (0.7)	
11. You need a hard toothbrush to clean your teeth			
Yes	108 (77.1)	7 (4.7)	*0*.000*
No	25 (17.9)	136 (91.3)	
Do n't know	7 (5.0)	6 (4.0)	
12. Dental floss is necessary to keep your teeth clean			
Yes	123 (87.9)	113 (75.8)	*0*.019*
No	12 (8.6)	20 (13.4)	
Do n't know	5 (3.6)	16 (10.7)	

Bold and italic values indicates statistically significant difference at *p* ≤ 0.05.

*Significant result, *p* < .05.

Regarding dental behavior, differences in habits were observed between groups. A smaller percentage of hearing-impaired individuals reported brushing their teeth once or twice daily compared to the individuals without hearing impairments (82.8% vs. 93.3%, *P* < 0.001). Although 91.3% of the HI group were able to brush their teeth independently, this figure was significantly lower than that of the individuals without hearing impairments, where 100% were able to do so (*P* = 0.001).

The use of dental floss, mouthwash, and fluoridated toothpaste was significantly lower among the individuals with hearing impairments (*P* < 0.05). For instance, only 5.8% of the HI group flossed daily compared to 16.8% of the individuals without hearing impairments (*P* = 0.009). Soda consumption was notably higher in the HI group (94.3% vs. 98.7% reporting no soda consumption; *P* < 0.001).

No significant differences were observed in the duration of brushing or the frequency of sweet consumption between the groups (*P* > 0.05). Detailed behavioral responses are provided in Table 3.

**Table 3:** Dental behavior among individuals with hearing impairments compared to those without hearing impairments.

Variable	Hearing impaired group, *n* (%)	Without hearing impairment group, *n* (%)	*P*-value
How often do you brush?			*0*.000*
Once or more	116 (82.8)	139 (93.3)	
Occasionally	24 (17.1)	10 (6.8)	
Brushing ability			*0*.001*
Completely without help	125 (91.3)	149 (100)	
Completely with help	12 (8.7)	0 (0)	
How often do you floss your teeth?			*0*.009*
Once or more a day	8 (5.8)	25 (16.8)	
Occasionally	128 (94.1)	124 (83.2)	
How often do you use mouthwash?			*0*.001*
Once or more a day	16 (11.4)	40 (26.8)	
Occasionally	124 (88.6)	109 (73.2)	
Do you use fluoridated toothpaste?			*0*.022*
Yes	85 (61.2)	113 (75.8)	
No	12 (8.6)	6 (4.0)	
Do n't know	42 (30.2)	30 (20.1)	
Frequency of eating sweets			0.354
Once or more a day	130 (97.1)	141 (94.6)	
Occasionally	4 (2.9)	8 (5.4)	
Frequency of drinking soda/day			*0*.000*
1–2 cans	132 (94.3)	147 (98.7)	
3–4 cans	0 (0)	2 (1.3)	
None	8 (5.7)	0 (0)	

Bold and italic values indicates statistically significant difference at *p* ≤ 0.05.

*Significant result, *p* < .05.

## Discussion

Hearing impairment is widely recognized as a barrier to effective communication, affecting social interaction, educational attainment, and overall quality of life (15). According to WHO estimates, 1 in 10 people globally will experience hearing loss by 2050 (3). In Jordan, the prevalence of significant hearing loss among infants was reported at 1.5% in 2014 (16), with a study conducted in 2024 estimating hearing impairment in only 0.06% of individuals aged 12 and above (17). This lower prevalence, compared to other nations, may reflect differences in screening programs or reporting methods. Notably, most participants in the present study had a severe hearing impairment, likely reflecting the recruitment from specialized centers catering to individuals with greater needs.

The prevalence of dental caries and caries experience, rate of gingival bleeding, and dental trauma in children with hearing impairment is high compared to figures observed in studies conducted among Jordanian schoolchildren with normal hearing (18).

Dental diseases and oral health can significantly impact the quality of life of individuals, as oral health is considered a crucial component of overall health and wellbeing (19). Although hearing impairment can reduce or eliminate the ability to receive and comprehend oral instructions and health promotion information, appropriate education on oral health hygiene using verbal and visual aids has been found to improve their oral health status and positively influence their perception of their quality of life (20).

Nevertheless, investigations into awareness, knowledge, and dental practices among individuals with hearing impairments have not been carried out in Jordan. Hence, this study aimed to investigate the oral health knowledge and dental practices amongst a cohort of Jordanian individuals with hearing impairments. Such an investigation was deemed necessary to scrutinize and analyze the gaps that exist in oral health knowledge that may need to be bridged through the implementation of applicable recommendations among people with hearing impairments.

The study found that hearing-impaired individuals exhibited comparable knowledge to individuals without hearing impairments in several key areas, including the role of plaque and bacteria in dental caries, the effects of sugary foods and carbonated beverages, and the relationship between oral and general health (21). This contrasts with a previous study conducted in Saudi Arabia, in which many hearing-impaired individuals were unaware of oral health basics or preventive care (22). Such findings suggest that in Jordan, efforts to raise awareness, perhaps through school-based programs or center-led initiatives, may have achieved some success. However, significant differences were observed in other domains. Individuals without hearing impairments demonstrated superior knowledge regarding toothbrushing.

The prevalence of regular tooth brushing for good oral health was higher among the Jordanian participants in the current study compared to a similar cohort of hearing-impaired individuals in other countries in the region, such as Saudi Arabia and Iran (22, 23).

In addition, most of the participants with hearing impairments reported that the primary reason for visiting a dentist was due to symptomatic oral disease. In contrast, a significant majority of the individuals without hearing impairments stated they visit the dentist even in the absence of a toothache. This lack of awareness regarding the importance of regular dental visits for asymptomatic individuals may contribute to a decline in their oral health. Without timely intervention, early-stage dental diseases are less likely to be detected, leading to more advanced dental issues with distressing symptoms and poor prognoses. This finding aligns with previous research by Mustafa et al. (2018) and Suma et al. (2011) (22, 24). Furthermore, irregular dentist visits have been associated with increased morbidity linked to dental caries (25). This pattern may also lead to a greater economic burden related to oral healthcare for this population.

Moreover, the hearing-impaired participants were less likely to recognize bleeding or swollen gums as signs of disease. These gaps highlight potential shortcomings in how oral health education reaches this population.

Interestingly, the hearing-impaired individuals showed greater awareness of the necessity of dental floss and correctly identified that healthy gums should not be red. This discrepancy, superior knowledge in some areas but poorer behaviors, warrants further exploration. One possible explanation is the role of visual learning tools, such as posters or video demonstrations, which may emphasize certain concepts more effectively than others (26). Studies from high-income countries also support the efficacy of visual and interactive education for hearing-impaired individuals (27, 28). Another possibility is prior exposure to tailored educational interventions at the centers where participants were recruited, although this could not be confirmed in the current study.

Despite having greater knowledge of flossing, the participants with hearing impairments used floss, mouthwash, and fluoridated toothpaste less frequently than their peers.

This gap between knowledge and practice underscores the critical insight that knowledge alone does not consistently motivate compatible dental health behaviors. Barriers such as affordability, limited product availability, and misconceptions about preventive care could contribute to this discrepancy (29, 30). Similar findings have been reported in other populations where awareness of preventive measures did not translate into action (31).

Confounding factors, including socioeconomic status, education level, and school type (mainstream vs. special education), may also influence these results. For example, lower family income and limited access to health services—more common among individuals with hearing impairments—could restrict opportunities to apply oral health knowledge in practice (32).

The contradiction between participants’ acknowledgment of sweets and soft drinks as detrimental and their continued high consumption highlights a broader challenge in public health: bridging the gap between awareness and sustained behavioral change (24, 33). Such patterns reinforce the importance of multifaceted interventions that address not only knowledge but also accessibility, affordability, and cultural attitudes toward oral health (26, 27).

Studies consistently demonstrate that hearing-impaired populations tend to have poorer oral hygiene, higher rates of caries, and worse oral health-related quality of life than their hearing peers (34, 35). However, the findings from this study suggest that targeted educational programs incorporating visual aids and culturally sensitive materials may help address these disparities.

Similar approaches in high-income countries have shown promising results, improving oral hygiene behaviors and reducing plaque and gingival scores (26, 28).

Although the current study employed a validated questionnaire, its reliance on literacy may have limited its applicability to all individuals with hearing impairments. Future studies should consider alternative data collection tools tailored to this population, such as sign language interviews or pictorial questionnaires, to enhance inclusivity. In addition, participants with hearing impairments were recruited through convenience sampling, which may have introduced selection bias. Moreover, while data were collected from multiple centers in Jordan, the sample size could be expanded in future research to improve generalizability and allow for adjustments based on age, gender, and socioeconomic status.

To our knowledge, this is the first study in Jordan to investigate oral health knowledge and practices among individuals with hearing impairments. The findings provide a valuable baseline for designing interventions to reduce oral health disparities and promote equitable access to dental care.

## Data Availability

The original contributions presented in the study are included in the article/Supplementary Material, further inquiries can be directed to the corresponding authors.
